# Long-term survival in a patient with primary refractory AML after salvage allogeneic hematopoietic transplantation and post-transplant localized irradiation and venetoclax maintenance: a case report

**DOI:** 10.3389/fonc.2023.1329858

**Published:** 2023-12-15

**Authors:** Yili Fan, Luyao Wang, Boxiao Chen, Jiawei Zhang, Luyu Yang, Xi Qiu, Huawei Jiang, Lei Zhu, Chao Wang, Yang Xu

**Affiliations:** ^1^ Department of Hematology, The Second Affiliated Hospital, Zhejiang University School of Medicine, Hangzhou, Zhejiang, China; ^2^ Department of Laboratory Medicine, the Second Affiliated Hospital, Zhejiang University School of Medicine, Hangzhou, China; ^3^ Department of Radiology, the Second Affiliated Hospital, Zhejiang University School of Medicine, Hangzhou, China

**Keywords:** primary refractory acute myeloid leukemia, allogeneic hematopoietic cell transplantation, relapse, radiotherapy, graft versus leukemia

## Abstract

For patients with primary refractory AML, allogeneic hematopoietic cell transplantation (allo-HCT) is considered the only curative approach. However, the therapeutic efficacy of salvage transplantation in the non-remission (NR) state remains controversial. We present a patient with primary refractory AML and concomitant central nervous system (CNS) leukemia, who received salvage allo-HCT, localized radiotherapy and venetoclax maintenance. Although he experienced systemic chronic graft-versus-host disease (cGVHD), he remained disease-free for 2 years. We propose that salvage transplantation is a feasible for primary refractory AML and discuss strategies to prevent relapse after allo-HCT, including maintenance therapy and donor lymphocyte infusion (DLI). Finally, we highlight the importance of radiotherapy, which can exert immunomodulatory effects to enhance immune responses against leukemia.

## 1 Introduction

Acute myeloid leukemia (AML) is a highly heterogeneous disease characterized by the abnormal accumulation of myeloblasts, with a 5-year survival rate of less than 30% following chemotherapy with or without hematopoietic cell transplantation (1). Primary refractory AML, defined as failure to achieve complete remission (CR) after 2 cycles of induction therapy, is associated with an extremely poor prognosis, with median OS of 6 months (2, 3). In addition, central nervous system (CNS) involvement in AML is common, especially among patients with hyperleukocytosis (>10×10^9^), or French-American-British (FAB) subgroups M4 and M5, which carry a poor prognosis with high relapse rates (4). Although salvage chemotherapy in combination with novel small molecule inhibitors targeting FLT3, IDH2 or Bcl2 can induce some responses, allogeneic hematopoietic cell transplantation (allo-HCT) remains the only curative treatment for refractory AML. Remission status prior to HCT has a significant impact on transplant outcome, where CR yields better outcomes than non-remission (NR) (5). However, primary refractory AML is unable to achieve CR before allo-HCT, but proceeding with salvage allo-HCT in selected patients could lead to a 3-year overall survival rate ranging from 14% to 40% (6). We report a case of a patient with primary refractory AML complicated with CNS leukemia, who achieved long-term survival with allo-HCT, post-HCT radiation and venetoclax maintenance.

## 2 Case presentation

**Figure 1:**
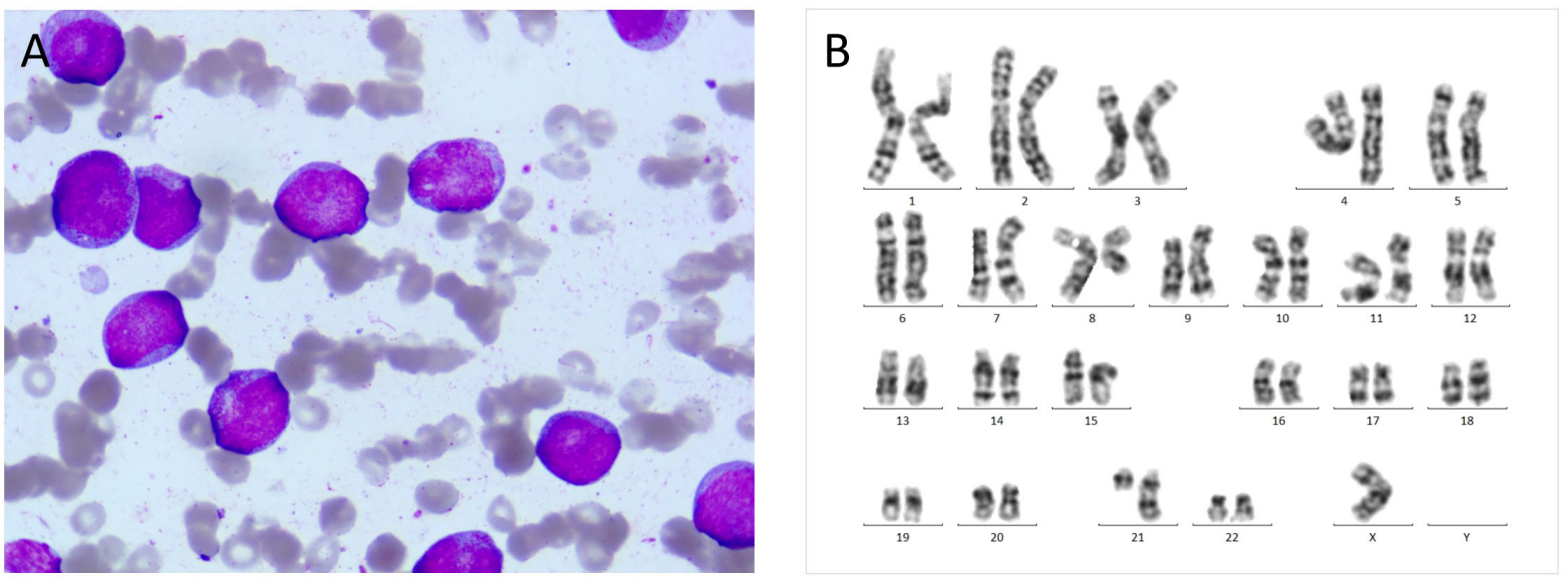
**(A)** At initial diagnosis, bone marrow aspiration revealed large myeloblasts with subtle nuclear irregularities and scattered cytoplasmic granules (HE, 1000×). **(B)** Chromosomal karyotype: 45, X, -Y, t (8;21) (q22; q22).

**Figure 2:**
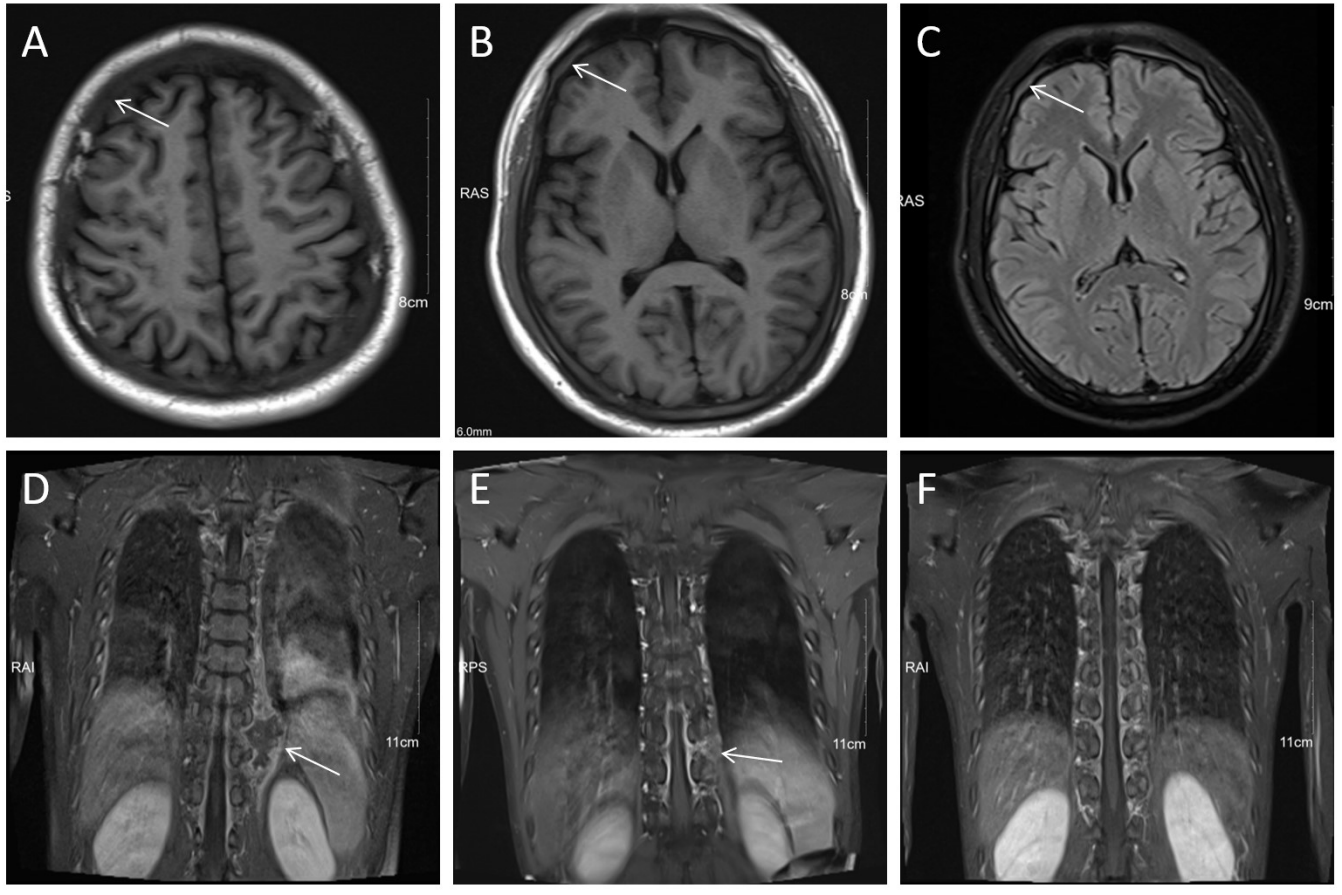
MRI revealed CNS involvement and extramedullary infiltration. **(A–C)** Diffuse hypointensity on T1WI and hyperintensity on T2-FlAIR of skull diploe (arrows), indicated leukocyte infiltration. **(D)** Soft tissue mass (arrow) adjacent to the left 9th-10th thoracic vertebrae on coronal T1WI+C. **(E, F)** Significant shrinkage of the soft tissue mass (arrow) of the thoracic vertebrae after localized radiotherapy.

In October 2020, a 24 years old man was referred to the emergency department because of fever and thrombocytopenia. Physical examination revealed right-sided deviation of the protruding tongue suggestive of hypoglossal nerve paralysis. Gingival hyperplasia and sternal tenderness were also noted. The complete blood count revealed a hemoglobin level of 76g/L, platelet count of 13×10^9^/L, and white blood cell count of 6.9×10^9^/L with 10% immature cells. Bone marrow aspiration showed 38.5% blast cells (**Figure 1A**). Immunophenotyping revealed that 61.8% of the immature cells were positive for HLA-DR, CD13, CD33, CD38, CD56, CD117, and MPO, while negative for CD10, CD20, CD22, CD61, cCD79a, and TdT. A cytogenetic analysis showed the presence of t (8;21) (**Figure 1B**), which resulted in the AML1/ETO fusion gene detected by PCR analysis. Genetic mutations in ASXL1, TP53, and c-kit genes were also identified. Hence, the diagnosis of acute myeloid leukemia with t (8;21) was confirmed. Enhanced MRI revealed slightly increased signals on both FLAIR and DWI sequences, indicating potential skull bone involvement (**Figures 2A–C**). Following two cycles of “3 + 7” chemotherapy with idarubicin and cytarabine, bone marrow blast count decreased to 4.0%; then a lumbar puncture was performed, revealing a CSF pressure of 220 mmH_2_O, with normal CSF analysis results. Intrathecal chemotherapy was administered, which led to resolution of neurological symptoms. However, in April 2021, a high-resolution CT of the chest revealed a new soft tissue mass adjacent to his thoracic vertebrae, most likely an extramedullary lesion (**Figure 2D**). He was treated with high-dose cytarabine or azacytidine in combination with venetoclax, but the extramedullary mass persisted and bone marrow relapse was documented. The patient’s condition worsened, with persistent fever and pulmonary infiltrates, despite aggressive use of broad-spectrum antibiotics. The underlying AML was thought to cause the fever. After an HLA-identical unrelated donor was found, the patient and his family opted for salvage allo-HCT. In June 2021, the patient underwent BuCy (busulfan and cyclophosphamide) conditioning before receiving peripheral blood stem cells with a CD34+ cell dose of 5.8×10^6^/kg, resulting in timely engraftment of neutrophils and platelets. A post-transplant MRI showed a significant reduction in soft tissue mass in the thoracic vertebrae, which was accompanied by a marked alleviation in back pain.

**Figure 3:**
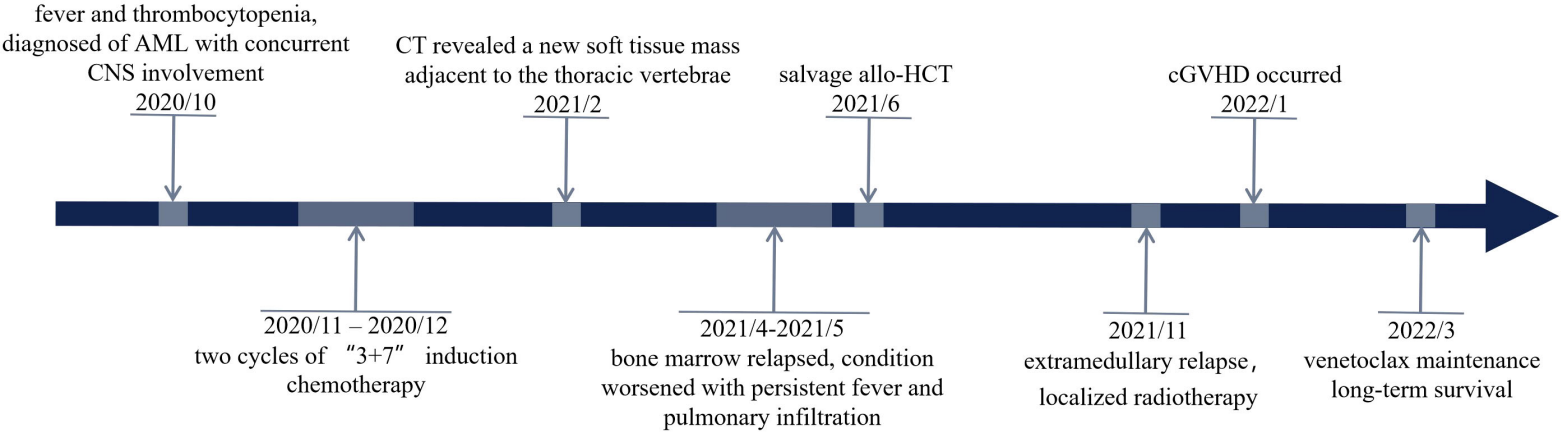
A timeline of the patient’s clinical courses.

To prevent AML relapse, immunosuppressive agents (ISTs) were discontinued month after HCT, and the patient had no manifestation of acute graft-versus-host disease (GVHD); he continued to receive maintenance of azacitidine and venetoclax. Bone marrow examination was negative for measurable minimal disease (MRD) by flow cytometry and PCR. After five months (November 2021), the paravertebral lesion recurred without any evidence of bone marrow relapse. The involved site radiation therapy (ISRT) effectively resolved the mass (**Figures 2E, F**). However, within one month after completion of radiotherapy, the patient developed acute skin injury in the treated area, with extensive skin peeling and hypopigmentation, followed by ulceration and crusting of the oral mucosa. These symptoms were consistent with systemic chronic GVHD (cGVHD), which were treated with low-dose corticosteroids and tacrolimus, combined with rehabilitation and nutritional support. The patient received 200 mg venetoclax daily for one year, resulting in an MRD-negative CR. The patient remained disease free for two years. The timeline of this case was summarized in **Figure 3**.

## 3 Discussion

While morphologic complete remission (CR) is typically preferred for transplant patients, accumulating evidence suggests that allo-HCT may provide long-term survival for a subset of patients with primary induction failure (6). In a study by Jabbour et al, the 3-year overall survival (OS) of 28 patients with primary refractory acute myeloid leukemia receiving allo-HCT was compared with that of 149 patients receiving salvage chemotherapy alone, with rates of 39% and 2%, respectively (7). A retrospective analysis conducted by the European Group for Blood and Marrow Transplantation (EBMT) indicated that there was no difference in 2-year OS rates between patients with primary refractory acute myeloid leukemia (AML) who received TBI-based and those who received busulfan-based conditioning, at 39.7% versus 35.3%, respectively (8). Another study demonstrated that allo-HCT could provide significant benefits to patients with refractory AML, regardless of the presence of concomitant extramedullary leukemia or leukemia burden during transplantation (9). Furthermore, the enhanced graft-versus-leukemia (GVL) effect can lead to reduced relapse rates among patients who develop cGVHD. Nevertheless, it is crucial to keep cGVHD at a mild to moderate severity level to prevent life-threatening complications (9).

Disease relapse remains challenging after salvage allo-HCT, with a relapse rate of about 40% (10, 11). Consequently, it is imperative to develop new strategies to improve disease control without causing excessive toxicity. Investigations have significantly increased with the introduction of various novel agents, including FLT3 inhibitors (midostaurin, sorafenib, gilteritinib), IDH inhibitors (ivosidenib, enasidenib), and hypomethylating agents (oral azacytidine, decitabine) (12). Venetoclax, a selective BCL-2 inhibitor, eliminates quiescent leukemic stem cells (LSCs), which are considered to be a major reservoir for relapse, by inhibiting their unique oxidative phosphorylation-dependent metabolic state (13). The Food and Drug Administration (FDA) has approved venetoclax in combination with HMA or low-dose cytarabine for the treatment of elderly or unfit patients with newly diagnosed AML. Several retrospective studies have reported improved outcomes of refractory/relapsed AML treated with venetoclax alone or in combination with other agents (14). Recently, venetoclax monotherapy has demonstrated safety, tolerability, and potential efficacy for maintenance therapy, improving OS and relapse-free survival in post-HCT patients at high risk of relapse (15). In addition, venetoclax can penetrate blood-brain barrier and potentially eradicate leukemia cells if measurable concentrations of venetoclax is present in the CSF (16). Consistently, our patient achieved an excellent outcome through venetoclax maintenance.

The most extensively studied interventions to alter the disease course after allo-HCT are the cessation of IST and donor lymphocyte infusion (DLI) (10). Nonetheless, due to the lack of prospective randomized trials (17), the effectiveness of preemptive DLI in MRD positive or mixed chimerism (MC) AML remains unclear. The 2016 EBMT Acute Leukemia Working Party (ALWP) guideline recommends cessation of all immunosuppression once MRD and/or escalating MC have been identified and DLI is initiated as early as D+100 in suitable high-risk patients while monitoring for GVHD (10). In addition to DLI, the advent of genetically engineered lymphocytes has provided novel treatment strategies, including multiple leukemia-associated antigen-targeting T cells and chimeric antigen receptor or T cell receptor-engineered cell therapies (18).

Treatment options for individuals with relapsed acute myeloid leukemia (AML) after HCT include palliative care, salvage chemotherapy, second transplantation, or cellular therapies, but long-term survival rates are low (19). However, our patient was treated with localized radiotherapy instead of systemic chemotherapy for extramedullary relapse. Radiotherapy (RT) provides a promising alternative to conventional treatments through induction of systemic immune response. Aside from damaging tumor cells directly, ionizing radiation can induce a significant anti-tumor immune response by affecting most stages of the cancer-immunity cycle, which includes amplifying the release and presentation of tumor antigens, immune cell activation and priming, increasing the density of tumor-infiltrating lymphocytes, improving the recognition of tumor cells by T cells, and augmenting anti-tumor effects (20). Based on this framework, the combination of RT and immunotherapies, such as immune checkpoint inhibitors, has been effective in treating various types of solid tumors (21). Therefore, radiotherapy can be utilized as an immunomodulator along with immunotherapy drugs or DLI to induce the graft-versus-leukemia (GVL) effect for treating AML. However, there are risks of developing GVHD for patients after allo-HCT.

## 4 Conclusion

Our case report describes a patient who had primary refractory AML and concurrent CNS leukemia. The patient achieved long-term survival after undergoing salvage allo-HCT, localized radiotherapy, and venetoclax maintenance despite cGVHD. We suggest that salvage allo-HCT represents a valid option for primary refractory AML and highlight the importance of post-transplant maintenance. The immunomodulatory effects of localized radiotherapy may have the potential to induce a GVL effect, but there is a risk of developing GVHD in post-HCT patients.

## Data availability statement

The original contributions presented in the study are included in the article/supplementary material. Further inquiries can be directed to the corresponding author.

## Ethics statement

The studies involving humans were approved by the Ethics Committee of the Second Affiliated Hospital of Zhejiang University School of Medicine. The studies were conducted in accordance with the local legislation and institutional requirements. Written informed consent for participation was not required from the participants or the participants’ legal guardians/next of kin in accordance with the national legislation and institutional requirements. Written informed consent was obtained from the individual(s) for the publication of any potentially identifiable images or data included in this article.

## Author contributions

YF: Writing – original draft, Data curation, Writing – review & editing, Formal analysis. LW: Methodology, Writing – review & editing. BC: Methodology, Writing – review & editing. JZ: Methodology, Writing – review & editing. LY: Methodology, Writing – review & editing. XQ: Writing – review & editing, Resources. HJ: Writing – review & editing, Investigation. LZ: Writing – review & editing, Visualization. CW: Writing – review & editing, Visualization, Validation. YX: Writing – review & editing, Investigation, Conceptualization, Data curation, Funding acquisition, Writing – original draft.
